# Is "Snapchat Dysmorphia" a Real Issue?

**DOI:** 10.7759/cureus.2263

**Published:** 2018-03-03

**Authors:** Kamleshun Ramphul, Stephanie G Mejias

**Affiliations:** 1 Department of Pediatrics, Shanghai Xin Hua Hospital Affiliated to Shanghai Jiao Tong University School of Medicine, Shanghai, People's Republic of China; 2 Department of Pediatrics, Robert Reid Cabral Children's Hospital Affiliated to the University Iberoamericana Unibe School of Medicine

**Keywords:** body dysmorphic syndrome, snapchat, instagram

## Abstract

It was observed that in early 2018, several newspapers raised a concern about the negative effects of social media applications, such as Snapchat and Instagram, on users related to the choice of plastic surgeries. Several plastic surgeons have shared their experiences whereby they encountered requests sounding similar to what a "filtered" Snapchat picture would look like, with one plastic surgeon even having a patient who actually produced a "filtered" image. There are several red flags to look out for in such patients, and proper management in those cases should include counseling and not plastic surgery.

## Editorial

In early 2018, multiple newspaper outlets published several articles questioning the current impact of social media applications, such as Snapchat and Instagram, related to the choice of plastic surgeries. The term "Snapchat Dysmorphia" was also coined, and we cannot help wonder how much these social applications are actually influencing the common man.

According to the American Psychiatric Association (APA) and Diagnostic and Statistical Manual of Mental Disorders (DSM-5), body dysmorphic disorder (BDD) is classified along the obsessive-compulsive Spectrum. Those suffering from BDD are preoccupied with at least one nonexistent or slight defect in physical appearance. This can lead them to think about the defect for at least one hour a day, therefore impacting their social, occupational, and other levels of functioning. The individual also should have repetitive and compulsive behaviors due to concerns arising from their appearances. This includes mirror checking and reassurance seeking among others [1]. Currently, one in 50 Americans suffers from BDD [2].

The two main applications in question included Snapchat and Instagram, both of which have 187-million and 600-million daily active users. These two applications provide filters that allow users to change their skin tone, soften fine lines and wrinkles, alter the size of their eyes, lips, and cheeks, and change various aspects of their physical appearance. Dr. Yagoda, a plastic surgeon, told the Huffington Post that he had observed many of his clients describing their desired changes, which corresponded to what the filters on these two applications could provide [3]. This claim was also supported by another plastic surgeon, Dr. Schulman. Renee Engeln, Professor of Psychology at Northwestern University, has also pointed out that the common man is losing perspectives on what he/she actually looks like due to these two social media applications [4]. The term "Snapchat Dysmorphia" was thus brought to life.

Another article published by The Independent reported a case whereby a plastic surgeon was requested to make a patient exactly like one of her "filtered" pictures. Dr. Esho politely declined and offered the patient some counseling help, which she eventually took. He also reported that the patient felt better with the help she received and is now making great progress. Moreover, he advised many physicians to look out for any red flags from patients who might have any underlying signs of body dysmorphia [5]. The outcomes of such plastic surgeries are usually unrealistic. This latest trend among millennials has been evolving for some time, but it is strange. Many factors may influence someone to opt for a surgical intervention to alter their appearance, consequently making psychological support a great help for the concerned person.

While the term "Snapchat Dysmorphia" might be too early to be brought into play, the risk of these patients turning to Snapchat and Instagram filters as a source of inspiration for their desired plastic surgeries is a big issue. There are already some ongoing legal issues about the use of Snapchat in the operating room by some plastic surgeons but none currently involving any patients accusing Snapchat of giving them a false perception of themselves yet. The proper code of ethics among plastic surgeons should be respected and an early detection of associated symptoms in such patients might help provide them with the appropriate counseling and help they need.
